# A Spontaneous Renal Calyceal Rupture Mimicking Physiologic Changes of Pregnancy and Other Common Pathologies

**DOI:** 10.7759/cureus.49006

**Published:** 2023-11-18

**Authors:** Karlene Vega-Figueroa, Pilar Silva-Melendez, Rocio Figueroa-Gonzalez, Andrea Colom-Diaz, Karina Gonzalez

**Affiliations:** 1 Obstetrics and Gynecology, Centro Medico Episcopal San Lucas, Ponce, PRI; 2 Obstetrics and Gynecology, Ponce Health Sciences University, Ponce, PRI

**Keywords:** second trimester renal rupture, preeclampsia, hydronephrosis in pregnancy, pregnancy, ruptured renal calyx

## Abstract

A spontaneous renal calyceal rupture in pregnancy is extremely rare and can be challenging to identify as its presentation can mimic other more common diagnoses, which can lead to a delay in management. Here, we describe an unusual case of renal calyceal rupture in a 24-year-old G2P0010 female with pregnancy at 26.5 weeks gestation age (WGA) who was admitted to the antepartum ward due to left flank pain and uterine contractions. A renal sonogram was performed, which revealed severe left hydronephrosis and the absence of the ipsilateral ureteral jet. Urinalysis was within normal limits, and her renal function was preserved. Laboratories were remarkable for elevated liver enzymes. Finally, an abdominopelvic MRI revealed the culprit, a calyceal rupture. Once the diagnosis was clear, a double J-stent was inserted using limited fluoroscopy with the goal of reducing intrarenal pressure and decreasing disease morbidity. The patient’s symptoms significantly improved after double J-stent placement and resolved the following day. The patient further developed preeclampsia with severe features, which has previously been documented to occur in pregnant patients with renal tract ruptures. The diagnosis of a renal calyceal rupture in pregnancy is not straightforward, in part because of a lack of awareness of this pathology. Nevertheless, early identification can prevent unnecessary interventions and adverse outcomes. Its diagnosis can be made with MRI, and its management with ureteral stent placement shouldn’t be delayed, and its association with preeclampsia should be further explored.

## Introduction

Although some urinary tract pathologies, such as hydronephrosis, are common given the physiologic changes that occur during pregnancy, spontaneous renal calyceal rupture is extremely rare, with less than 40 reported cases [1]. Calyceal rupture in pregnancy can be particularly challenging to identify as its symptoms, primarily of flank pain, can mimic other more common diagnoses such as pyelonephritis, renal colic, or even preterm labor, leading to delay in appropriate management. Renal tract ruptures usually occur with an underlying obstructive cause, such as nephrolithiasis and renal masses, but they have been reported to occur without their presence. Also, with the current information available, it can be inferred that pregnancy can be a risk factor for renal tract ruptures on its own [2].

Additionally, renal tract rupture has been seen to occur in pregnant patients who later develop preeclampsia [3]. Given the paucity of data regarding renal tract rupture in pregnancy, a direct association between the two pathologies cannot be formally established. However, one can theorize that the pathophysiology of renal tract rupture and preeclampsia development could be intertwined [4].

## Case presentation

**Figure 1:**
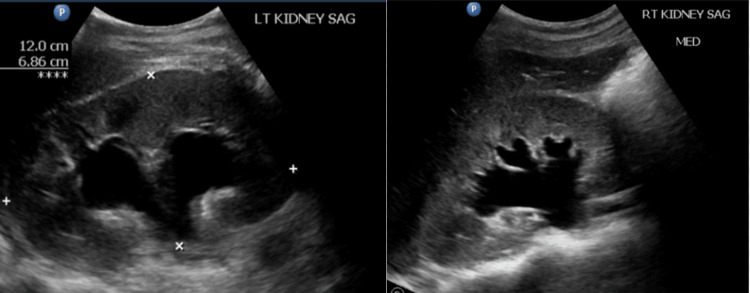
Renal ultrasound revealing severe bilateral hydronephrosis

A 24-year-old G2P0010 female with pregnancy at 26.5 weeks gestational age (WGA) was admitted to the antepartum ward due to left flank pain and painful regular uterine contractions. The patient had been previously hospitalized at 25.3 WGA for similar symptoms and was treated for preterm labor with a steroid course for fetal lung maturation, tocolysis, magnesium sulfate for fetal neuroprotection, and antibiotics for group B *Streptococcus* prophylaxis. However, at that time, no additional pathologies other than physiologic hydronephrosis on renal ultrasound were identified for which the patient was discharged home after completing treatment. On this occasion, the patient denied symptoms of vaginal bleeding, fever, chills, nausea, vomiting, dysuria, or hematuria and reported adequate fetal movements. Abdominal examination was unremarkable except for left costovertebral angle tenderness. Fetal assessment with biophysical profile and external fetal monitoring was normal. Pelvic examination revealed only physiologic leukorrhea, and the cervical os (opening of the cervix) was 2 cm dilated and 60% effaced, unchanged from previous admission. Urinalysis showed no hematuria or leukocyturia. The complete metabolic panel was remarkable for elevated liver enzymes with aspartate aminotransferase (AST) at 131 u/l and alanine aminotransferase (ALT) at 144 u/l, and anemia with hemoglobin at 10.5 g/dL. Kidney function tests were within normal limits, with blood urea nitrogen (BUN) at less than 5 mg/dL and creatine at 0.51 mg/dL. A renal ultrasound was subsequently performed, which revealed bilateral severe hydronephrosis, more prominent on the left side, with the absence of the left ureteral jet (Figure 1).

**Figure 2:**
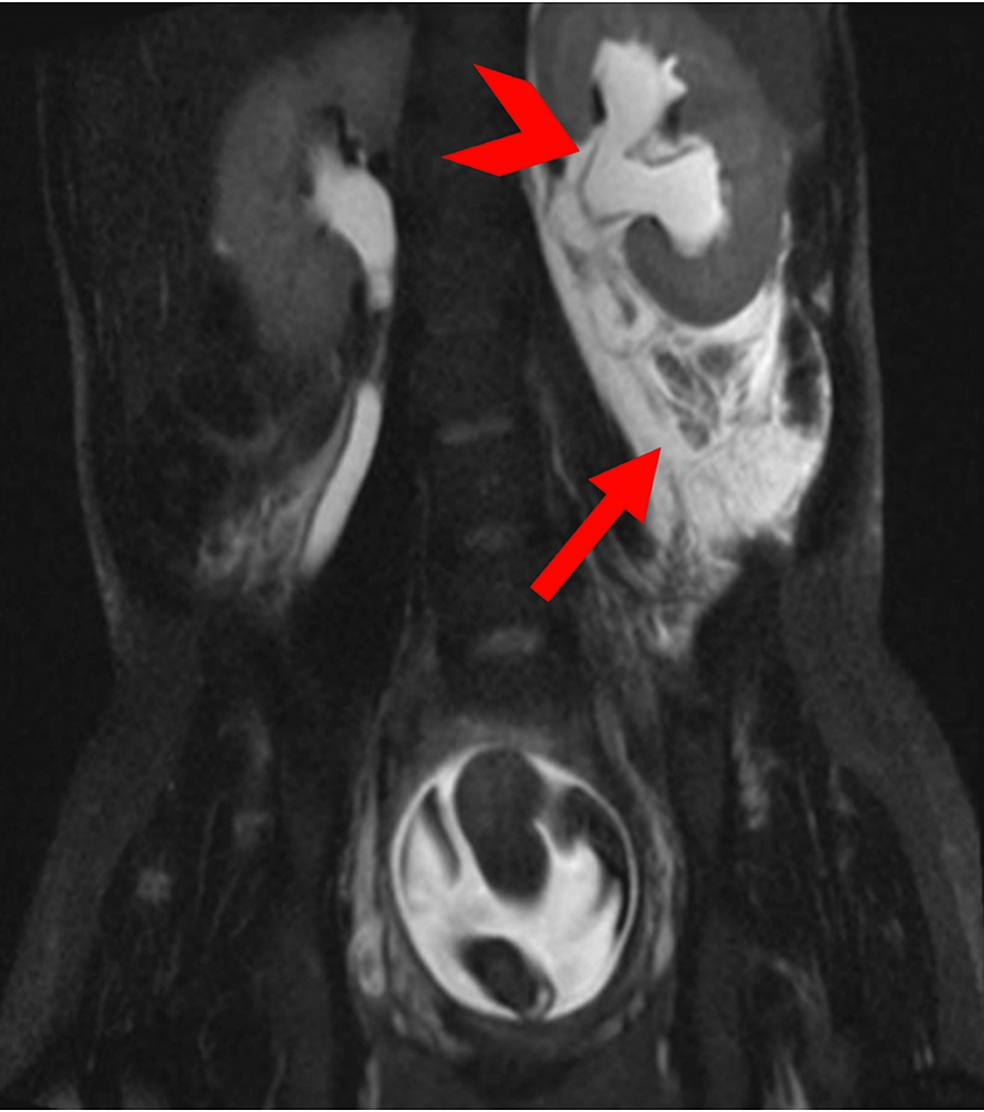
Abdominopelvic MRI (coronal view) The image shows the left hydroureter (arrowhead) and fluid extravasation surrounding the left kidney (arrow).

There was no additional evidence of an obstructing pathology, such as a mass or nephrolithiasis. A renal calyceal rupture and urinoma diagnoses were confirmed via abdominopelvic MRI without contrast upon visualization of a large amount of left perinephric fluid (Figure 2). 

**Figure 3:**
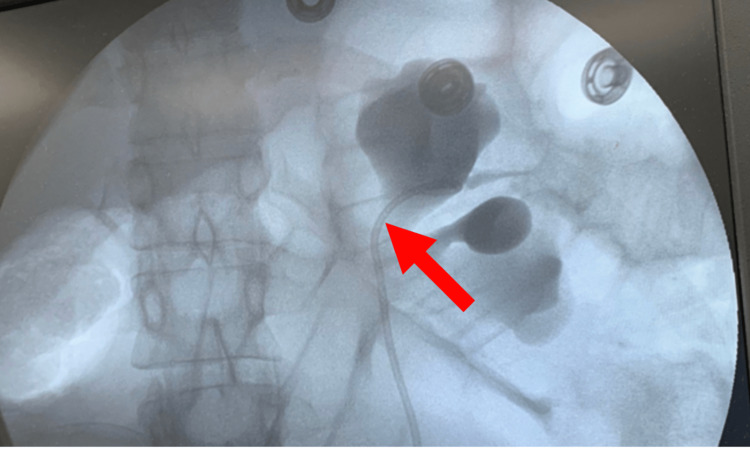
Limited fluoroscopy imaging The image shows the location of the left ureteral stent (arrow).

Initial concerns for preterm delivery and fetal lung maturity were addressed during the patient’s prior admission, where she received betamethasone. While awaiting confirmatory MRI results, the patient’s left flank pain was managed with morphine, resulting in moderate pain improvement. Once the diagnosis was clear, the primary concern was reducing intrarenal pressure to avoid the possibility of urine leakage into the retroperitoneum, urinoma formation, and preterm labor. The patient underwent placement of a left double J-stent, inserted using limited fluoroscopy to reduce radiation to the patient and fetus, which would be left in place for the duration of the pregnancy (Figure 3). After double J-stent placement, the patient’s symptoms improved significantly the following day. Repeat laboratories performed three days after the intervention showed a decreasing trend in liver enzymes, with an AST of 79 u/l and an ALT of 126 u/l. The patient was discharged home 72 hours post-stent placement in stable condition with double J-stent in place and antibiotic therapy with cephalexin 500 mg orally every 12 hours for 10 days.

Labor was induced at 34 6/7 weeks gestation due to preeclampsia with severe features due to elevated blood pressures over 140/90 on more than two occasions over four hours apart, proteinuria (protein creatinine ratio of 1.99), unremitting headache, and scotoma. Liver enzymes at this time were within normal limits (AST: 22 u/L, ALT: 13 u/L). That same day the patient vaginally delivered a healthy baby girl (Apgar of 8 at 1 minute and 9 at 5 minutes) with a birth weight of 2720 g. The delivery was complicated by postpartum hemorrhage, which required blood transfusions but resulted in no further complications. The patient was lost to follow-up, but at 18 weeks postpartum and six and a half months after stent placement, she arrived at the emergency room with left flank discomfort. Evaluation with abdominopelvic CT scan revealed a calcified left double J-stent in place, which was able to be removed by the Urologist without any complications.

## Discussion

Physiologic changes mediated by a rise in a variety of hormones during pregnancy are known to significantly affect the renal system via increased circulating blood volume, renal perfusion, glomerular filtration rate, and vasodilation [5]. These changes predispose pregnant women to several renal changes such as hydronephrosis. Progressive ureteric mechanical obstruction throughout pregnancy causes dilation of renal calyces and physiologic hydronephrosis, leading to an increased risk of urinary stasis and pyelonephritis. It has been studied that the maximal incidence of hydronephrosis is reached at 28 WGA, consistent with the growing uterus further obstructing the ureters and making pregnancy a risk factor for the described pathology. 

One comparable case report describes a spontaneous renal forniceal rupture in a 26-year-old primigravid woman at 23 WGA, presenting with flank pain and costovertebral angle tenderness. In this patient’s case, the rupture was initially managed symptomatically, but the patient returned with urinoma four days later, requiring the placement of a nephrostomy tube [6]. This complication highlights the need for immediate diagnosis and management to promptly resolve the disease and prevent further complications such as preterm labor [7].

Delays in the diagnosis of a renal calyceal rupture, are probably due to its clinical presentation appearing as renal tract physiologic changes of pregnancy, such as hydronephrosis, and other renal tract pathologies, such as nephrolithiasis. In our case, urinalysis and urinary function tests were within normal limits, and interestingly, the patient’s liver enzymes were elevated upon arrival and demonstrated a decreasing trend after treatment. Such findings suggest that retroperitoneal urinoma expansion possibly led to liver irritation and elevated liver enzymes. Prompt reduction of intrarenal pressure with the placement of a double J-stent is crucial to reduce urine leakage into the retroperitoneum, leading to urinoma formation, sepsis, severe kidney injury, nephrostomy tube placement, and even preterm labor and delivery [8]. Yet, it is important to perform and educate patients that proper urologic follow-up after pregnancy should take place in order to avoid stent calcification, migration, stone formation, or blockage. Recommended replacement or removal time should be within one month to six months of placement [9]. 

Another interesting detail in our case was the development of preeclampsia further in the pregnancy, which could have been an independent occurrence. Yet, another case report of renal tract rupture in pregnancy also concluded in a patient being induced due to preeclampsia [3]. Such a finding could mean there is an association between both pathologies. Notably, Reuter et al. reported a theory on how compression of the left renal vein by the growing uterus could lead to preeclampsia via increased intrarenal pressure (renal compartment syndrome), activation of the renin-angiotensin-aldosterone system, and the release of endothelin-1 [4]. Said theory could explain how compression of the renal vein by the pressure exerted by a urinoma contributes to preeclampsia development. Yet, considering the complex pathophysiology of preeclampsia, which is thought to start at the beginning of pregnancy, one could argue that the development of preeclampsia could have been the cause of renal rupture. However, the pathophysiology of preeclampsia is complex and likely multifactorial for which a clear association between renal calyx rupture and preeclampsia development cannot be accurately established and should be further studied.

## Conclusions

The diagnosis of a renal calyceal rupture in pregnancy is challenging and may be overlooked in favor of more common renal physiologic changes in pregnancy. Nevertheless, early identification and suspicion once more common pathologies have been ruled out is crucial to preventing unnecessary interventions and adverse maternal-fetal outcomes. Typically, in non-pregnant patients, its diagnosis is confirmed using a CT scan with IV contrast. In pregnancy, ultrasonography and MRI have proven sufficient for diagnosis, avoiding patient and fetal exposure to radiation. Once identified, prompt reduction of intrarenal pressure with ureteral stent placement should be performed due to its safety and effectiveness. It can remain in place throughout the pregnancy to reduce the risk of retroperitoneal urine leakage and further complications. Finally, an increased risk of developing preeclampsia in patients with renal tract rupture, and vice versa, should be considered.
